# Prognostic value of sleep apnea and nocturnal hypoxemia in patients with decompensated heart failure

**DOI:** 10.1002/clc.23319

**Published:** 2020-01-22

**Authors:** Yuhui Huang, Yunhong Wang, Yan Huang, Mei Zhai, Qiong Zhou, Xuemei Zhao, Pengchao Tian, Shiming Ji, Chen Zhang, Yuhui Zhang, Jian Zhang

**Affiliations:** ^1^ Heart Failure Center, State Key Laboratory of Cardiovascular Disease Fuwai Hospital, National Center for Cardiovascular Diseases, **Chinese Academy of Medical Sciences** and Peking Union Medical College Beijing People's Republic of China

**Keywords:** acute decompensated heart failure, nocturnal hypoxemia, sleep apnea

## Abstract

**Background:**

Nocturnal hypoxemia is an important factor underlying the impact of sleep apnea on heart failure. It remains unclear whether nocturnal hypoxemia has a greater prognostic value in acute decompensated heart failure (ADHF) compared with the frequency of sleep apnea.

**Hypothesis:**

Nocturnal hypoxemia might be better than the frequency of sleep apnea in predicting the outcomes in ADHF.

**Methods:**

Sleep studies were prospectively performed during an ADHF hospitalization from January 2015 to December 2017. Sleep apnea was defined as the apnea‐hypopnea index (AHI) ≥15/h. The severity of nocturnal hypoxemia was determined by the percentage of time with saturation below 90% (T90%). The endpoint was the first event of all‐cause death, heart transplantation, implantation of left ventricular assist device, unplanned hospitalization for worsening heart failure, acute coronary syndrome, significant arrhythmias, or stroke.

**Results:**

Of 382 patients, 189 (49.5%) had sleep apnea. The endpoint incidence did not differ between AHI categories (≥15/h vs <15/h: 52.4% vs 44.6%, log rank *P* = .353), but did between T90% categories (≥3.6% vs <3.6%: 54.5% vs 42.4%, log rank *P* = .023). Multivariate Cox regression analysis showed that T90% was independently associated with the endpoint (hazard ratio [HR] 1.008, 95% confidence interval [CI] 1.001‐1.016, *P* = .033), whereas AHI was not; the risk of the endpoint increased by 40.8% in patients with T90% ≥3.6% (HR 1.408, 95%CI 1.030‐1.925, *P* = .032).

**Conclusion:**

Nocturnal hypoxemia had a greater prognostic value in ADHF than the frequency of sleep apnea.

## 1 INTRODUCTION

Sleep apnea, typically categorized as predominantly obstructive (OSA) or central (CSA), is highly prevalent in both acute decompensated heart failure (ADHF)^1^, ^2^ and chronic stable heart failure.^3^, ^4^ Sleep apnea is responsible for multiple cardiovascular pathophysiological changes in heart failure, such as myocardial ischemia,^5^ increased pulmonary arterial pressure,^6^ and abnormal cardiac electrophysiological activities,^7^, ^8^ based on complex mechanisms, including nocturnal hypoxemia, increased sympathetic activity, enhanced renin‐angiotensin‐aldosterone system, and chronic inflammation.^9^, ^10^ It has been reported that sleep apnea, generally scored by the apnea‐hypopnea index (AHI), might be an independent risk factor of adverse outcomes in heart failure.^1^, ^2^, ^11^ However, AHI has been questioned as a prognostic predictor of heart failure in some studies.^12^, ^13^ AHI is only a metric reflecting the frequency of apneas and hypopneas during sleep and does not take the lengths of apneas and hypopneas into consideration on its own definition. Therefore, more importance should be attached to detailed characteristics of sleep apnea.

Nocturnal hypoxemia, as a composite consequence of apneas and hypopneas, might better represent the adverse effects of nocturnal respiratory events in heart failure. Gottlieb et al reported that increased hemodynamic stress in heart failure was related to the percentage of time with saturation below 90% (T90%), but not to the AHI.^14^ Evidence also suggested that nocturnal hypoxemia appeared to be more robust to predict outcomes in stable chronic heart failure compared with AHI.^11^, ^15^ However, it is unclear whether nocturnal hypoxemia is better than AHI in predicting the outcomes in ADHF. Therefore, in the present study, we aimed to compare AHI and several parameters of nocturnal hypoxemia in evaluating the prognosis in hospitalized heart failure patients.

## 2 METHODS

### 2.1 Patients

This single‐center, prospective, observational study was performed in Heart Failure Center, Fuwai Hospital. From January 2015 to December 2017, patients with ADHF were consecutively enrolled, including both new‐onset heart failure and decompensation of chronic heart failure. ADHF was diagnosed based on symptoms/signs of fluid overload and/or hypoperfusion, and appropriate additional investigations such as chest X‐ray, electrocardiogram, N‐terminal pro‐brain natriuretic peptide (NT‐proBNP), and echocardiography according to the European Society of Cardiology Guidelines.^16^ The exclusive criteria were as follows: age <18 or >80 years; any coronary event within the previous 3 months or at the time of enrollment, namely, acute coronary syndrome (ACS), percutaneous coronary invention, coronary artery bypass grafts; implantation of pacemaker, implantable cardioverter defibrillation (ICD), or cardiac resynchronization therapy within the previous 3 months; heart valvular surgeries within the previous 3 months; stroke within the previous 6 months; dialysis; chronic obstructive pulmonary disease; acute myocarditis or infective endocarditis; significant uncorrected valvular heart disease; malignancy; pregnancy; diagnosed sleep apnea, or previously receiving any type of positive pressure ventilation or oxygen therapy. Patients were also excluded if they were admitted to hospital for cardiovascular interventions and surgeries. The study protocol conformed to the Declaration of Helsinki and was approved by the institutional review board of Fuwai Hospital. Individual informed consents were signed.

### 2.2 Sleep study

Patients received sleep studies by means of Apnealink Plus (Resmed, Ltd, Martinsried, Germany) from 22:00 to 6:00 after an initial improvement of heart failure during the hospitalization period by intensive therapy. Patients undergoing sleep study were requested to relieve from edema and lie in a supine position without dyspnea under room air. Sleep studies were not done on patients who were hemodynamically unstable, had nocturnal dyspnea, needed oxygen supplement or ventilation. Nasal airflow amplitude and oxygen saturation were measured by a nasal flow pressure cannula and a finger pulse oximeter, respectively. The recorded data were analyzed by two‐step method. First, the data were analyzed automatically by software, Apnealink Version 10.20. Then the recordings were manually reanalyzed by a sleep specialist who was blinded to the clinical status of patients. In recordings, only time periods with both sufficient airflow and saturation signals were considered valid recording time. We only took account those sleep studies with a minimum 4‐hour valid recording time. Apnea was defined as breathing amplitude decreased by ≥90% for ≥10 seconds. Hypopnea was defined as breathing amplitude decreased by ≥30% lasting for ≥10 seconds, accompanied by a ≥3% drop in oxygen saturation.^17^ AHI was defined as the total number of apneas and hypopneas per hour. Sleep apnea was defined as AHI ≥15/h. Oxygen desaturation index (ODI) was defined as the total number of desaturation events where oxygen saturation decreased by ≥3% per hour. The mean saturation (meanSO_2_), the minimal saturation (minSO_2_), and T90% during sleep were also recorded.

### 2.3 Blood samples and echocardiography

Blood samples were routinely collected for every patient. We examined a series of blood parameters, including NT‐proBNP, hemoglobin, serum creatinine (SCr), blood urea nitrogen, potassium, sodium, glycated hemoglobin, total cholesterol, and low‐density lipoprotein cholesterol. The renal function was evaluated by eGFR (mL/min/1.73 m^2^) based on SCr using modification of diet in renal disease (MDRD) equation. Renal dysfunction was defined as eGFR <60 mL/min/1.73 m^2^. Echocardiography was performed using ultrasound system (Vivid E9; GE, Norway) on admission.

### 2.4 Follow‐up and endpoint

The enrolled patients were systematically followed up every 3 months by outpatient reviews or telephone calls after discharge until December 31, 2018. Follow‐up was terminated when death, heart transplantation, or implantation of left ventricular assist device (LVAD) occurred. The endpoint was defined as the first event of death from any cause, heart transplantation, LVAD implantation, unplanned hospitalization for worsening heart failure, ACS, significant arrhythmias, and stroke. Significant arrhythmia event was defined as sustained ventricular tachycardia, ventricular fibrillation of asystole. Information of the adverse events was obtained from the medical records for those patients who were followed up at our hospital. For those patients who were not followed up at our hospital, detailed information was obtained by telephone calls with patients' families and local medical institutions they were admitted to if necessary. Data regarding the adverse events were collected and determined by two blinded cardiologists.

### 2.5 Statistical analysis

Continuous variables were presented as mean ± SD or median with interquartile range (IQR) as appropriate, while categorical variables were expressed as frequency and percentage. Baseline characteristics were compared with Student's *t* test or Mann‐Whitney *U* test for continuous variables, and chi‐square test or Fisher's exact test for categorical variables. The impact of each sleep study parameters on the time to the endpoint was assessed by Kaplan‐Meier analysis using log‐rank test. The thresholds of sleep study parameters were determined by the median values except for AHI. Factors associated with the endpoint were determined using univariate Cox regression analysis, including age, gender, BMI, coronary artery disease, hypertension, diabetes mellitus, dyslipidemia, atrial fibrillation, renal dysfunction, NYHA class, mean arterial blood pressure (MAP) at discharge, NT‐proBNP, LVEF, medications prescribed at discharge (ie, angiotensin converting enzyme inhibitor [ACEI] /angiotensin receptor blocker [ARB], β‐blocker, spironolactone, calcium channel blocker, and statin) and sleep study parameters. Variables with *P* < .10 in univariate analysis were included in a multivariate Cox regression analysis to identify the independent risk factors of the endpoint based on stepwise backward selection using a likelihood ratio (*P* > .5 for exclusion). Sleep study parameters were included in multivariate analysis irrespective of their significance in univariate analysis. Because of potential correlation between sleep study parameters, each tested parameter was analyzed separately in multivariate analysis. Hazard ratios (HR) and 95% confidence intervals (CI) were calculated. A two‐tailed *P* < .05 was considered statistically significant. All data were analyzed using SPSS version 23.0 (IBM corporation, Armonk, New York).

## 3 RESULTS

**Figure 1:**
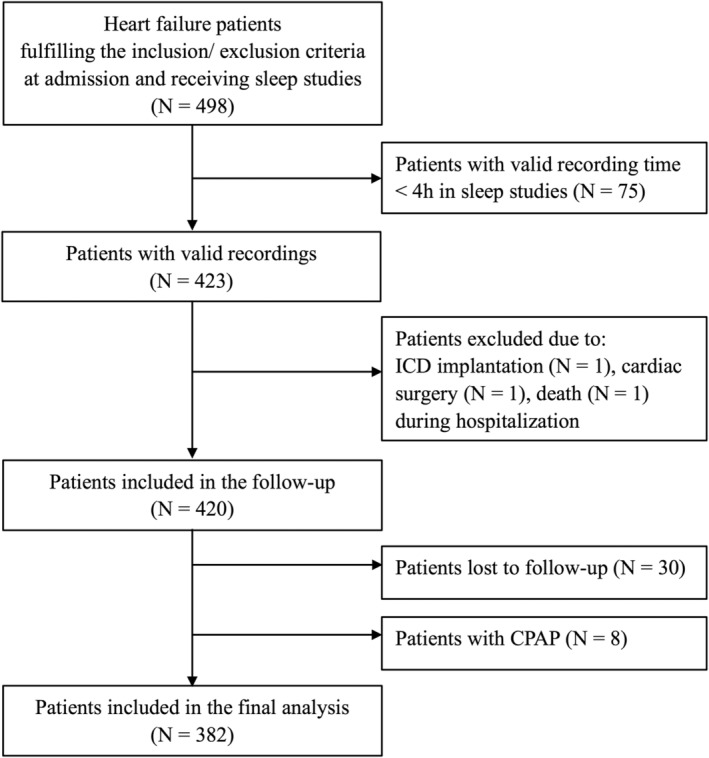
Study flow chart. ICD, implantable cardioverter defibrillation

A total of 420 patients who met the predefined inclusion/exclusion criteria were followed up systematically after discharge. Follow‐up was completed in 390 (92.9%) patients. We excluded eight patients who were treated with continuous positive airway pressure ventilation to minimize the effect of ventilation therapy on the prognosis (Figure 1). Of 382 patients included in the final analysis, 189 (49.5%) had sleep apnea (AHI ≥15/h). Patients with AHI ≥15/h were characterized by more males, higher BMI, a higher prevalence of hypertension, higher NT‐proBNP, and lower LVEF. Patients with AHI ≥15/h also tended to have severe NYHA class and higher blood pressure (Table S1). The median value of ODI, meanSO_2_, minSO_2_, T90% of the whole population were 19.0/h, 95.0%, 79.0%, and 3.6%, respectively. Patients with T90% ≥3.6% had severer NYHA class, higher NT‐proBNP, and lower LVEF at baseline (Table S2).

The median follow‐up was 19.7 months (IQR, 14.4‐25.2 months) and the maximum follow‐up was 45.5 months. During the follow‐up, 72 patients died from any cause, 17 patients underwent heart transplantation. Regarding the predefined endpoint, a total of 185 adverse events occurred. In detail, 51 patients died from any cause (42 with cardiovascular death, 1 with noncardiovascular death, 8 with sudden death), 15 underwent heart transplantation, 104 had hospitalizations for worsening heart failure, 3 had ACS, 4 had significant arrhythmias with successful resuscitation (3 with subsequent implantation of ICD), and 8 had stroke. Compared to those without the endpoint, patients with the endpoint were characterized by older age, lower BMI, a higher prevalence of renal dysfunction, higher NYHA class, lower blood pressure, higher NT‐proBNP, lower LVEF, less ACEI/ARB, and more diuretics. There were no differences of AHI and AHI categories between patients with and without the endpoint. T90% was significantly higher in patients with the endpoint (Table 1).

**Table 1:** Baseline characteristics of patients with and without clinical event

	No event (N = 197)	Event (N = 185)	*P*
Age (years)	51 ± 16	57 ± 13	.001
Male (N, %)	149 (75.6)	136 (73.5)	.634
BMI (Kg/m^2^)	26.2 ± 5.0	24.8 ± 4.8	.004
Current smoker (N, %)	41 (20.8)	25 (13.5)	.059
Coronary artery disease (N, %)	50 (25.4)	54 (29.2)	.403
Hypertension (N, %)	103 (52.3)	82 (44.3)	.120
Diabetes mellitus (N, %)	50 (25.4)	55 (29.7)	.341
Dyslipidemia (N, %)	90 (45.7)	73 (39.5)	.219
Renal dysfunction (N, %)	41 (20.8)	77 (41.6)	<.001
Atrial fibrillation (N, %)	55 (27.9)	67 (36.2)	.082
Cardiac electronic device implantation (N, %)	10 (5.1)	20 (10.8)	.037
NYHA III/IV (N, %)	143 (72.6)	164 (88.6)	<.001
SBP on admission (mm Hg)	125 ± 22	117 ± 21	<.001
DBP on admission (mm Hg)	76 ± 15	71 ± 13	.001
MAP on admission (mm Hg)	93 ± 15	86 ± 14	<.001
Heart rate on admission (bpm)	80 ± 19	78 ± 15	.190
Awake SO_2_ in supine position (%)	96.8 ± 2.1	96.9 ± 2.0	.977
NT‐proBNP (pg/mL)	1427.0 (529.0, 3216.0)	4027.0 (1791.0, 9732.0)	<.001
Hemoglobin (g/L)	147 ± 21	142 ± 22	.019
Sodium (μmol/L)	138.8 ± 3.8	137.9 ± 3.7	.019
Potassium (μmol/L)	3.9 ± 0.5	4.0 ± 0.5	.126
Creatinine (μmol/L)	83.9 (72.2, 97.5)	96.5 (79.7, 120.2)	<.001
eGFR (mL/Kg/1.73 m^2^)	80.7 ± 27.4	67.1 ± 23.4	<.001
BUN (mmol/L)	6.5 (5.2, 8.4)	7.9 (6.3, 10.0)	<.001
HbA1c (mmol/L)	6.4 ± 1.0	6.6 ± 1.1	.040
Total cholesterol (mmol/L)	4.1 ± 1.0	3.9 ± 1.0	.174
LDL‐C (mmol/L)	2.6 ± 0.8	2.5 ± 0.8	.575
LVEF (%)	38.0 (29.0, 50.0)	32.0 (25.0, 48.0)	.011
SBP at discharge (mm Hg)	113 ± 13	107 ± 14	<.001
DBP at discharge (mm Hg)	68 ± 9	65 ± 11	.038
MAP at discharge (mm Hg)	83 ± 9	80 ± 10	.001
Heart rate at discharge (bpm)	71 ± 12	72 ± 10	.655
Medication at discharge			
ACEIs/ARBs (N, %)	148 (75.1)	106 (57.3)	<.001
β‐blockers (N, %)	182 (92.4)	168 (90.8)	.579
Spironolactone (N, %)	147 (74.6)	143 (77.3)	.541
Digoxin (N, %)	109 (55.3)	112 (60.5)	.303
Diuretic (N, %)	176 (89.3)	178 (96.2)	.010
Calcium channel blockers (N, %)	15 (7.6)	10 (5.4)	.383
Statins (N, %)	97 (49.2)	75 (40.5)	.088
Sleep study			
AHI (/h)	13.6 (5.7, 28.1)	16.3 (7.2, 31.9)	.233
Sleep apnea (N, %)	90 (45.7)	99 (53.5)	.126
ODI (/h)	18.4 (9.2, 32.9)	19.6 (11.8, 33.3)	.264
MeanSO_2_ (%)	94.5 ± 2.5	93.9 ± 3.0	.073
MinSO_2_ (%)	76.8 ± 11.4	74.5 ± 11.7	.058
T90% (%)	2.1 (0.2, 12.6)	5.5 (0.6, 22.6)	.008

Abbreviations: ACEI, angiotensin converting enzyme inhibitor; AHI, apnea‐hypopnea index; ARB, angiotensin receptor blocker; BMI, body mass index; BUN, blood urea nitrogen; DBP, diastolic blood pressure; eGFR, estimated glomerular filtration rate; HbA1c, glycosylated hemoglobin; LDL‐C, low density lipoprotein cholesterol; LVEF, left ventricular ejection fraction; MAP, mean arterial blood pressure; meanSO_2_, mean oxygen saturation; minSO_2_, minimal oxygen saturation; NT‐proBNP, N‐terminal pro‐brain natriuretic peptide; NYHA, New York Heart Association; ODI, oxygen desaturation index; SBP, systolic blood pressure; T90%, the percentage of time with oxygen saturation below 90%.

**Figure 2:**
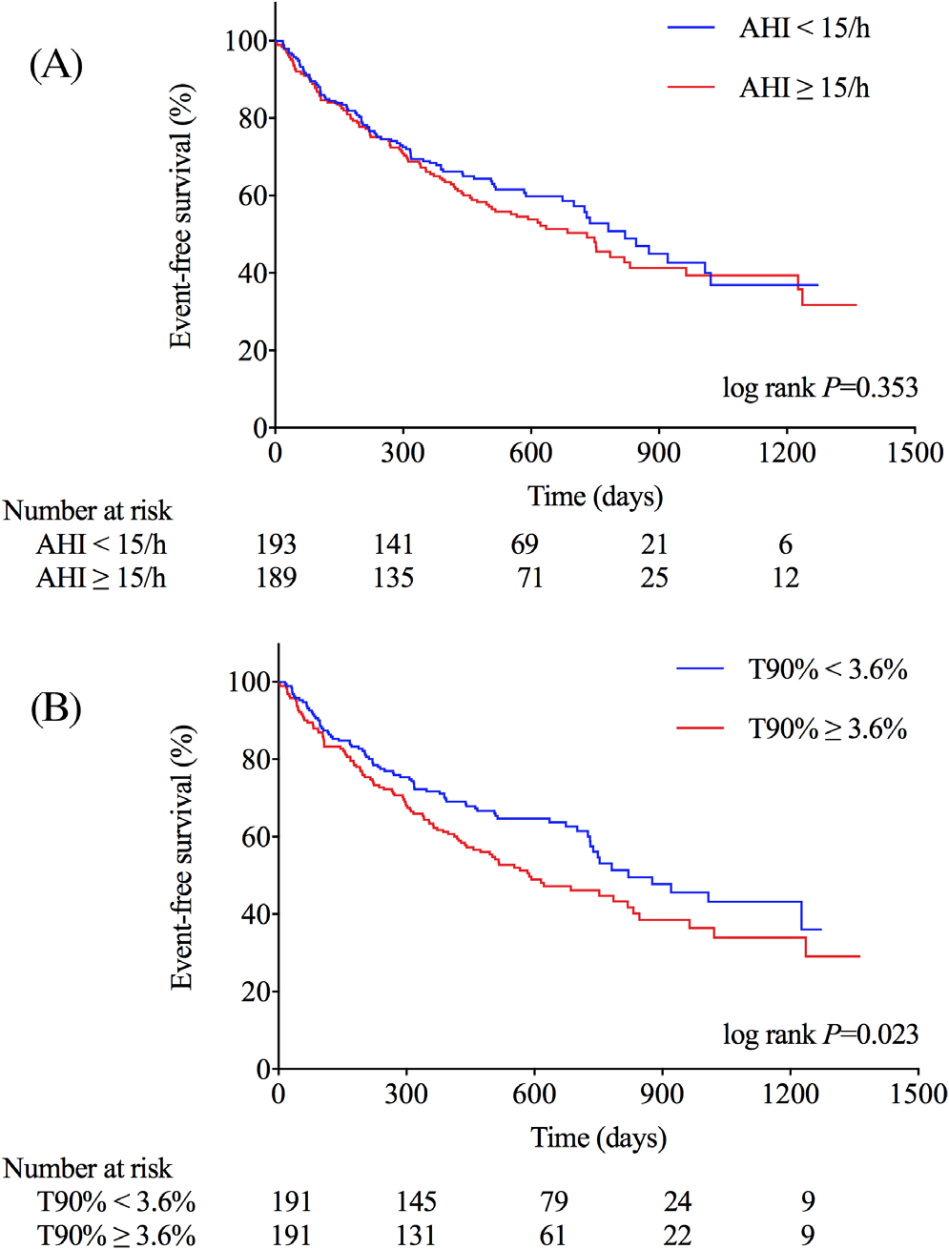
Kaplan‐Meier curves for event‐free survival according to the categories of AHI (A) or T90% (B). AHI, the apnea‐hypopnea index; T90%, the percentage of time with oxygen saturation below 90%

The Kaplan‐Meier analysis showed the incidence of the endpoint did not differ between AHI categories (≥15/h vs <15/h: 52.4% vs 44.6%, χ^2^ = 0.862, log rank *P* = .353; Figure 2A), ODI categories (≥19.0/h vs < 19.0/h, 50.8% vs 46.0%, χ^2^ = 0.461, log rank *P* = .497), meanSO_2_ categories (<95.0% vs ≥95.0%, 51.1% vs 45.9%, χ^2^ = 0.630, log rank *P* = .428), or minSO_2_ categories (<79.0% vs ≥79.0%, 54.7% vs 42.2%, χ^2^ = 2.933, log rank *P* = .087). Patients with T90% ≥3.6% had a significantly higher incidence of the endpoint than those with T90% <3.6% (54.5% vs 42.4%, χ^2^ = 5.137, log rank *P* = .023; Figure 2B).

Univariate Cox analysis showed T90% was associated with the endpoint (HR 1.007, 95%CI 1.000‐1.014, *P* = .049), the risk of the endpoint increased by 39.7% in patients with T90% ≥3.6% compared to those with T90% <3.6% (HR 1.397, 95%CI 1.045‐1.869, *P* = .024). However, neither AHI (HR 1.003, 95%CI 0.944‐1.012, *P* = .491) nor AHI ≥15/h (HR 1.147, 95%CI 0.859‐1.532, *P* = .354) showed significant association with the endpoint (Table S3). Univariate analysis also showed that age, BMI, hypertension, atrial fibrillation, renal dysfunction, NYHA class (III/IV), NT‐proBNP, LVEF, MAP, ACEI/ARB, and diuretics were associated with the endpoint with a statistical significance of *P* < .10. The above variables along with sleep study parameters were included in multivariate Cox analysis (Table S4).

In stepwise multivariate Cox regression analysis, the effect of T90% on the prognosis was still statistically significant (HR 1.008, 95%CI 1.001‐1.016, *P* = .033). The risk of the endpoint increased by 40% in patients with T90% ≥3.6% (HR 1.408, 95%CI 1.030‐1.925, *P* = .032; Table 2). The results of multivariate analysis also demonstrated that the risk of the endpoint was associated with the level of minSO_2_ (HR 0.985, 95%CI 0.973‐0.997, *P* = .017), the risk of the endpoint was 39.5% higher in patients with minSO_2_ <79.0% than those with minSO_2_ ≥79.0% (HR 1.395 95%CI 1.038‐1.876, *P* = .028; Table 3). MeanSO_2_ was significant statistically as a continuous variable in multivariate analysis (HR 0.950, 95%CI 0.905‐0.998, *P* = .040), but not as a categorical variable (<95.0% vs ≥95.0%). However, AHI and ODI were not independent predictors of adverse outcomes in multivariate Cox analysis either as continuous or categorical variables.

**Table 2:** Prognostic role of T90% in stepwise multivariate Cox regression analysis

	Multivariate Cox regression analysis	
	HR (95%CI)	*P*
*T90% as a continuous variable*		
Age (per 10 years increase)	1.144 (1.035‐1.264)	.008
BMI (per 5 kg/m^2^ increase)	0.630 (0.444‐0.893)	.009
NYHA III/IV (yes vs no)	1.672 (1.047‐2.669)	.031
NT‐proBNP (per 500 pg/mL increase)	1.027 (1.018‐1.037)	<.001
MAP at discharge (per 10 mm Hg increase)	0.797 (0.673‐0.944)	.009
T90% (per 1% increase)	1.008 (1.001‐1.016)	.033
*T90% as a categorical variable*		
Age (per 10 years increase)	1.138 (1.030‐1.258)	.011
BMI (per 5 kg/m^2^ increase)	0.636 (0.450‐0.900)	.011
NYHA III/IV (yes vs no)	1.640 (1.026‐2.622)	.039
NT‐proBNP (per 500 pg/mL increase)	1.028 (1.019‐1.038)	<.001
MAP at discharge (per 10 mm Hg increase)	0.795 (0.671‐0.943)	.008
T90% ≥3.6% (yes vs no)	1.408 (1.030‐1.925)	.032

Abbreviations: BMI, body mass index; CI, confidence interval; HR, hazard ratio; MAP, mean atrial blood pressure; NT‐proBNP, N‐terminal pro‐brain natriuretic peptide; NYHA, New York Heart Association; T90%, the percentage of time with oxygen saturation below 90%.

**Table 3:** Prognostic role of minSO_2_ in multivariate analysis

	Multivariate Cox regression analysis	
	HR (95%CI)	*P*
*MinSO*_*2*_ *as a continuous variable*		
Age (per 10 years increase)	1.147 (1.037‐1.269)	.008
BMI (per 5 kg/m^2^ increase)	0.652 (0.468‐0.908)	.011
NYNA III/IV (yes vs no)	1.774 (1.112‐2.831)	.016
NTproBNP (per 500 pg/mL increase)	1.028 (1.019–1.038)	<.001
MAP at discharge (per 10 mm Hg increase)	0.780 (0.660‐0.922)	.004
MinSO_2_ (per 1% increase)	0.985 (0.973‐0.997)	.017
*MinSO*_*2*_ *as a categorical variable*		
Age (per 10 years increase)	1.147 (1.037‐1.268)	.007
NYNA III/IV (yes vs no)	1.782 (1.117‐2.845)	.015
NT‐proBNP (per 500 pg/mL increase)	1.028 (1.018–1.037)	<.001
MAP at discharge (per 10 mm Hg increase)	0.789 (0.667‐0.934)	.006
ACEI/ARB at discharge (yes vs no)	0.665 (0.489‐0.906)	.010
MinSO_2_ <79.0% (yes vs no)	1.395 (1.038‐1.876)	.028

Abbreviations: ACEI, angiotensin converting enzyme inhibitor; ARB, angiotensin receptor blocker; BMI, body mass index; CI, confidence interval; HR, hazard raito; MAP, mean atrial blood pressure; MinSO_2_, the minimal oxygen saturation; NT‐proBNP, N‐terminal pro‐brain natriuretic peptide; NYHA, New York Heart Association.

## 4 DISCUSSION

In this study, we compared the impact of AHI and parameters of nocturnal hypoxemia on the prognosis in patients with decompensated heart failure. The results showed that the nocturnal hypoxemia, calculated by the percentage of time with saturation below 90% (T90%), rather than the frequency of apneas and hypopneas, was an independent predictor of adverse outcomes in decompensated heart failure.

The proportion of sleep apnea (AHI ≥15/h) in the present study was smaller than previous studies.^1^, ^2^ Compared with those studies, BMI in the present study was lower. As AHI is correlated with BMI,^18^, ^19^ the relatively lower BMI might partly explain the lower prevalence of sleep apnea. Another important factor contributing to the lower prevalence of sleep apnea was that the patients received intensive therapy of heart failure before sleep studies. Fluid overload and congestion are important characteristics of ADHF. In ADHF, fluid redistribution from the lower extremities to the lungs in supine position would aggravate pulmonary congestion, which further elicits reflex hyperventilation and provokes hypocapnia, resulting in the stimulation of CSA. The fluid shift also facilitates OSA by exacerbating the obstruction of upper airways.^20^, ^21^ Most patients in the study were relieved from congestion and edema after intensive therapy before sleep studies, as a consequence, sleep apnea was probably alleviated.

AHI might not be the best metric to determine the severity of sleep apnea in heart failure. As mainly reflecting the frequency of apneas and hypopneas during sleep on its own definition, AHI does not consider the durations of apneas and hypopneas. As a consequence, AHI cannot differentiate between apneas and hypopneas with the same number but different durations. Moreover, the lengths of apneas and hypopneas are dependent on cardiac function.^22^ The greater the extent of cardiac dysfunction, the longer apneas and hypopneas will be. As a result, the total number of apneas and hypopneas is potentially limited in heart failure and the severity of sleep apnea, determined by AHI, is consequently underestimated.

The result of our study demonstrated that some parameters representative of nocturnal hypoxemia (ie, T90% and minSO_2_) were better than AHI in predicting adverse outcomes in ADHF, which was consistent with some previous studies in stable chronic heart failure. Oldenburg et al^11^ reported that the time with saturation below 90% (T90) was a more robust predictor of all‐cause mortality than AHI or the phenotypes of sleep apnea in a large cohort with a long‐term follow‐up. The risk of death increased by 16.1% (HR 1.161, *P* < .001) for every 1‐hour increase in T90 after adjustment for other important predictors. In a multivariable Cox regression model including both AHI and T90, only T90 was the dominant predictor of all‐cause mortality (HR 1.157, *P* < .001). Another prospective study also compared the prognostic value of nocturnal desaturation parameters and AHI in chronic stable heart failure. The results showed T90 and minSO_2_ were independently associated with a higher risk of all‐cause death, heart transplantation, and LVAD implantation (HR for T90 1.36, *P* = .007; HR for minSO_2_ 1.29, *P* = .008). However, neither AHI nor sleep apnea (AHI ≥15/h) showed significant association with the prognosis.^15^ In addition, minSO_2_ was also demonstrated a more robust association with fatal or resuscitated sudden cardiac death than AHI.^23^

These findings suggested that nocturnal hypoxemia might better represent the detrimental effects of sleep apnea than the frequency of apneas and hypopneas in heart failure. It was reported that T90%, rather than AHI, predicted the elevations in brain natriuretic peptide,^14^ indicating nocturnal hypoxemia might be an important factor underlying the impact of sleep apnea on acute hemodynamic stress in heart failure. Another study found that cardiac norepinephrine spillover was correlated with a reduced oxygen saturation, but not with the AHI,^24^ indicating increased sympathetic activity was more associated with nocturnal hypoxemia. Overall, nocturnal hypoxemia might be a better measure representative of adverse effects of sleep apnea than AHI, explaining why it was better than the frequency of apneas and hypopneas in predicting the prognosis in ADHF. Additionally, AHI value does not accurately reflect the severity of nocturnal hypoxemia. T90 values of zero or close to zero were frequently observed in combination with a wide range of AHI values,^11^ which indicated that apneas or hypopneas, especially those with shorter durations, might not necessarily cause relevant hypoxemia and be effective to induce pathophysiological changes.

The present study suggested that nocturnal hypoxemia is a potential treating target of sleep apnea in heart failure. Although positive airway pressure ventilation is recommended in some clinical conditions of sleep apnea^25^ and has been proven to improve hemodynamic condition and cardiac function,^6^, ^26^, ^27^ its effect on the prognosis of heart failure patients still remains controversial, especially in those with predominantly CSA.^28^, ^29^ Considering the cost and the tolerance, nocturnal oxygen (NOT) appeared to be an alternative of positive airway pressure ventilation during sleep. NOT was shown to improve AHI, nocturnal hypoxemia, left ventricular function, and quality of life in heart failure patients with CSA or OSA.^30^, ^31^ However, concerns regarding detrimental hyperoxia‐induced cardiovascular effects also have been raised in heart patients with oxygen therapy, including reduction in cardiac output, increase in systemic vascular resistance.^32^, ^33^ Finally, the impact of long‐term use of NOT on the prognosis of patients with heart failure and sleep apnea remains unknown. Well‐designed randomized clinical trials are needed to determine the prognostic effect of oxygen supplement in treating sleep apnea in heart failure. Recently, LOFT‐HF trial (NCT03745898) has been approved by National Institute of Health (NIH), which might help to identify the effect of oxygen therapy on the prognosis of patients with heart failure and sleep apnea. To note, several novel therapeutic methods of sleep apnea have been developed, such as phrenic nerve stimulation and oral appliance. Phrenic nerve stimulation has been shown effective to improve AHI and hypoxemia in patients with CSA.^34^ A randomized controlled trial has been registered to assess the efficacy of an oral appliance for sleep‐disordered breathing and cardiac function in patients with heart failure.^35^ These novel therapeutic approaches might provide new insight into treatment of sleep apnea.

We acknowledged there were several limitations in the present study. First, the relatively small sample size and short follow‐up might decrease the generalizability of the prognostic value of AHI and the parameters of nocturnal hypoxemia. Second, we used a portable screening device rather than the full polysomnography to determine AHI. Theoretically, AHI obtained from the portable device is probably underestimated because it calculates the recording time rather than the actual sleep time and does not take arousals into consideration. Third, pulmonary congestion might be a potential factor to influence blood oxygen saturation. In the study, patients received intensive therapy of heart failure before sleep studies. All patients undergoing sleep studies were requested to lie in a supine position without dyspnea under room air and relieve from edema. Although we did not perform specific imaging examination to assess pulmonary edema at the time of sleep studies, clinical status of patients indicated that pulmonary edema was alleviated. Forth, we did not consider the influence of treatment for sleep apnea on the prognosis during the follow‐up. We excluded the effect of ventilation therapy from the analysis because the percentage of patients treated with nocturnal ventilation was quite small in the present study. The low level of awareness of sleep apnea and the high expenditure of ventilator might explain the low percentage of ventilation usage to some extent. Besides, it remains controversial over the prognostic effect of positive airway pressure ventilation in sleep apnea.^29^, ^36^, ^37^ Considering the results of the study, further research is required to examine the prognostic influences of oxygen therapy in patients with heart failure and sleep apnea.

## 5 CONCLUSION

This study demonstrated that nocturnal hypoxemia was more predictive of adverse outcomes in decompensated heart failure than the frequency of sleep apnea. Prospective studies should be conducted to determine the effect of oxygen therapy on the prognosis of heart failure and sleep apnea.

## CONFLICT OF INTEREST

The authors declare no potential conflict of interests.

## Supporting information


**Table S1** Baseline characteristics between patients with AHI < 15/h and ≥ 15/h

**Table S2** Baseline characteristics between patients with T90% < 3.6% and ≥ 3.6%

**Table S3** Prognostic role of sleep study parameters in univariate analysis

**Table S4** Factors selected in stepwise multivariate Cox regression analysis

**Table S5**A, B, and C shows the process of multivariate Cox regression analysis, which are considered for review but not for publication
