# Supplementary material for: Prognostic value of sleep apnea and nocturnal hypoxemia in patients with decompensated heart failure
Source: Clin Cardiol. 2020 Jan 22;43(4):329–37. doi: 10.1002/clc.23319 (PMC7144483; doi:10.1002/clc.23319)
Supplement: Supplementary file 1 — Table S1 Baseline characteristics between patients with AHI < 15/h and ≥ 15/h [file CLC-43-329-s001.docx]

Supplemental Table 1 Baseline characteristics between patients with AHI < 15/h and ≥ 15/h

|  | AHI < 15/h (N=193) | AHI ≥ 15/h (N=189) | *P* |
| --- | --- | --- | --- |
| Age (years) | 54±16 | 54±14 | 0.759 |
| Male (N, %) | 122 (63.2) | 163 (86.2) | <0.001 |
| BMI (Kg/m^2^) | 24.4±4.4 | 26.7±5.2 | <0.001 |
| Current smoker (N, %) | 28 (14.5) | 38 (20.1) | 0.148 |
| Coronary artery disease (N, %) | 49 (25.4) | 55 (29.1) | 0.415 |
| Hypertension (N, %) | 81 (42.0) | 104 (55.0) | 0.011 |
| Diabetes mellitus (N, %) | 46 (23.8) | 59 (31.2) | 0.106 |
| Dyslipidemia (N, %) | 82 (42.5) | 81 (42.9) | 0.942 |
| Renal dysfunction (N, %) | 63 (32.6) | 55 (29.1) | 0.454 |
| Atrial fibrillation (N, %) | 61 (31.6) | 61 (32.3) | 0.889 |
| Cardiac electronic device implantation (N, %) | 15 (7.8) | 15 (7.9) | 0.952 |
| NYHA Ⅲ/Ⅳ (N, %) | 148 (76.7) | 159 (84.1) | 0.067 |
| SBP on admission (mmHg) | 120±22 | 122±22 | 0.601 |
| DBP on admission (mmHg) | 72±14 | 76±14 | 0.004 |
| MAP on admission (mmHg) | 88±15 | 91±15 | 0.044 |
| Heart rate on admission (bpm) | 77±17 | 81±17 | 0.012 |
| Awake SO_2_ in supine position (%) | 96.9±2.0 | 96.8±2.2 | 0.723 |
| NT-proBNP (pg/mL) | 1854.0 (630.0, 4780.4) | 2798.0 (1150.0, 7152.0) | 0.003 |
| Hemoglobin (g/L) | 141.7±21.1 | 147.1±21.2 | 0.014 |
| Sodium (μmol/L) | 137.9±4.0 | 138.8±3.5 | 0.019 |
| Potassium (μmol/L) | 4.0±0.5 | 4.0±0.5 | 0.441 |
| Creatinine (μmol/L) | 86.3 (71.0, 108.3) | 91.2 (79.4, 109.7) | 0.031 |
| eGFR (mL/Kg/1.73m^2^) | 75.0±28.5 | 73.2±24.1 | 0.522 |
| BUN (mmol/L) | 7.2 (5.3, 9.1) | 7.4 (5.9, 9.3) | 0.135 |
| HbA1c (mmol/L) | 6.4±1.1 | 6.5±1.1 | 0.157 |
| Total cholesterol (mmol/L) | 4.0±0.9 | 4.0±1.0 | 0.884 |
| LDL-C (mmol/L) | 2.5±0.8 | 2.5±0.8 | 0.983 |
| LVEF (%) | 39.0 (29.0, 57.0) | 32.0 (26.0, 43.0) | < 0.001 |
| SBP at discharge (mmHg) | 111±14 | 111±13 | 0.689 |
| DBP at discharge (mmHg) | 65±10 | 68±10 | 0.022 |
| MAP at discharge (mmHg) | 80±10 | 82±10 | 0.074 |
| Heart rate at discharge (bpm) | 71±10 | 72±12 | 0.412 |
| Medication at discharge |  |  |  |
| ACEIs/ARBs (N, %) | 115 (59.6) | 139 (73.5) | 0.004 |
| β-blockers (N, %) | 175 (90.7) | 175 (92.6) | 0.498 |
| Spironolactone (N, %) | 138 (71.5) | 152 (80.4) | 0.041 |
| Digoxin (N, %) | 94 (48.7) | 127 (67.2) | <0.001 |
| Diuretic (N, %) | 171 (88.6) | 183 (96.8) | 0.002 |
| Calcium channel blockers (N, %) | 14 (7.3) | 11 (5.8) | 0.571 |
| Statins (N, %) | 90 (46.6) | 82 (43.4) | 0.524 |
| Sleep study |  |  |  |
| AHI (/h) | 6.2 (3.7, 10.5) | 30.2 (21.2, 40.5) | < 0.001 |
| ODI (/h) | 10.0 (6.0, 16.0) | 32.9 (24.4, 42.9) | < 0.001 |
| MeanSO_2_ (%) | 95.1±2.1 | 93.3±3.1 | < 0.001 |
| MinSO_2_ (%) | 76.9±12.3 | 74.4±10.8 | 0.035 |
| T90% (%) | 0.6 (0.2, 4.3) | 11.2 (3.4, 25.0) | < 0.001 |

ACEI, angiotensin converting enzyme inhibitor; AHI, apnea-hypopnea index; ARB, angiotensin receptor blocker; BMI, body mass index; BUN, blood urea nitrogen; DBP, diastolic blood pressure; eGFR, estimated glomerular filtration rate; HbA1c, glycosylated hemoglobin; LDL-C, low density lipoprotein cholesterol; LVEF, left ventricular ejection fraction; MAP, mean arterial blood pressure; meanSO_2_, mean oxygen saturation; minSO_2_, minimal oxygen saturation; NT-proBNP, N-terminal pro-brain natriuretic peptide; NYHA, New York Heart Association; ODI, oxygen desaturation index; SBP, systolic blood pressure; T90%, the percentage of time with oxygen saturation below 90%
