# Supplementary material for: Prognostic value of sleep apnea and nocturnal hypoxemia in patients with decompensated heart failure
Source: Clin Cardiol. 2020 Jan 22;43(4):329–37. doi: 10.1002/clc.23319 (PMC7144483; doi:10.1002/clc.23319)
Supplement: Supplementary file 2 — Table S2 Baseline characteristics between patients with T90% < 3.6% and ≥ 3.6% [file CLC-43-329-s002.docx]

Supplemental Table 2 Baseline characteristics between patients with T90% < 3.6% and ≥ 3.6%

|  | T90% < 3.6% (N=191) | T90% ≥ 3.6% (N=191) | *P* |
| --- | --- | --- | --- |
| Age (years) | 53±15 | 55±15 | 0.265 |
| Male (N, %) | 135 (70.7) | 150 (78.5) | 0.078 |
| BMI (Kg/m^2^) | 24.2±4.3 | 26.9±5.2 | <0.001 |
| Current smoker (N, %) | 31 (16.2) | 35 (18.3) | 0.588 |
| Coronary artery disease (N, %) | 47 (24.6) | 57 (29.8) | 0.250 |
| Hypertension (N, %) | 87 (45.5) | 98 (51.3) | 0.260 |
| Diabetes mellitus (N, %) | 46 (24.1) | 59 (30.9) | 0.136 |
| Dyslipidemia (N, %) | 80 (41.9) | 83 (43.5) | 0.756 |
| Renal dysfunction (N, %) | 52 (27.2) | 66 (34.6) | 0.121 |
| Atrial fibrillation (N, %) | 54 (28.3) | 68 (35.6) | 0.124 |
| Cardiac electronic device implantation (N, %) | 11 (5.8) | 19 (9.9) | 0.128 |
| NYHA Ⅲ/Ⅳ (N, %) | 142 (74.3) | 165 (86.4) | 0.003 |
| SBP on admission (mmHg) | 120±22 | 121±22 | 0.621 |
| DBP on admission (mmHg) | 73±14 | 75±14 | 0.137 |
| MAP on admission (mmHg) | 89±15 | 91±15 | 0.251 |
| Heart rate on admission (bpm) | 76±15 | 81±19 | 0.007 |
| Awake SO_2_ in supine position (%) | 97.4±1.5 | 96.3±2.4 | <0.001 |
| NT-proBNP (pg/mL) | 1822.0 (602.0, 4127.5) | 2930.0 (1219.0, 8230.0) | <0.001 |
| Hemoglobin (g/L) | 144±20 | 145±23 | 0.710 |
| Sodium (μmol/L) | 138.5±4.2 | 138.2±3.3 | 0.374 |
| Potassium (μmol/L) | 3.9±0.5 | 4.0±0.5 | 0.176 |
| Creatinine (μmol/L) | 85.9 (73.9, 99.6) | 92.6 (77.2, 117.3) | 0.008 |
| eGFR (mL/Kg/1.73m^2^) | 76.5±25.9 | 71.8±26.8 | 0.081 |
| BUN (mmol/L) | 6.8 (5.3, 8.6) | 7.6 (6.0, 9.6) | 0.001 |
| HbA1c (mmol/L) | 6.3±1.0 | 6.6±1.1 | 0.011 |
| Total cholesterol (mmol/L) | 4.1±0.9 | 4.0±1.0 | 0.286 |
| LDL-C (mmol/L) | 2.6±0.8 | 2.5±0.8 | 0.670 |
| LVEF (%) | 38.0 (29.0, 53.5) | 32.0 (26.0, 46.0) | 0.001 |
| SBP at discharge (mmHg) | 111±14 | 110±13 | 0.460 |
| DBP at discharge (mmHg) | 66±10 | 67±10 | 0.079 |
| MAP at discharge (mmHg) | 81±10 | 82±9 | 0.378 |
| Heart rate at discharge (bpm) | 71±10 | 72±12 | 0.487 |
| Medication at discharge |  |  |  |
| ACEIs/ARBs (N, %) | 120 (62.8) | 134 (70.2) | 0.129 |
| β-blockers (N, %) | 172 (90.1) | 178 (93.2) | 0.268 |
| Spironolactone (N, %) | 139 (72.8) | 151 (79.1) | 0.151 |
| Digoxin (N, %) | 94 (49.2) | 127 (66.5) | 0.001 |
| Diuretic (N, %) | 167 (87.4) | 187 (97.9) | <0.001 |
| Calcium channel blockers (N, %) | 13 (6.8%) | 12 (6.3) | 0.863 |
| Statins (N, %) | 92 (48.2) | 80 (41.9) | 0.217 |
| Sleep studies |  |  |  |
| AHI (/h) | 7.5 (4.2, 15.6) | 27.8 (14.3, 40.0) | <0.001 |
| Sleep apnea (N, %) | 49 (25.7%) | 140 (73.3%) | <0.001 |
| ODI (/h) | 11.8 (6.1, 18.5) | 31.3 (20.1, 42.2) | <0.001 |
| MeanSO_2_ (%) | 95.8±1.3 | 92.6±2.9 | <0.001 |
| MinSO_2_ (%) | 78.9±11.0 | 72.5±11.3 | <0.001 |
| T90% (%) | 0.4 (0.0, 1.1) | 18.1 (8.9, 30.8) | <0.001 |

ACEI, angiotensin converting enzyme inhibitor; AHI, apnea-hypopnea index; ARB, angiotensin receptor blocker; BMI, body mass index; BUN, blood urea nitrogen; DBP, diastolic blood pressure; eGFR, estimated glomerular filtration rate; HbA1c, glycosylated hemoglobin; LDL-C, low density lipoprotein cholesterol; LVEF, left ventricular ejection fraction; MAP, mean arterial blood pressure; meanSO_2_, mean oxygen saturation; minSO_2_, minimal oxygen saturation; NT-proBNP, N-terminal pro-brain natriuretic peptide; NYHA, New York Heart Association; ODI, oxygen desaturation index; SBP, systolic blood pressure; T90%, the percentage of time with oxygen saturation below 90%
