# Supplementary material for: Prognostic value of sleep apnea and nocturnal hypoxemia in patients with decompensated heart failure
Source: Clin Cardiol. 2020 Jan 22;43(4):329–37. doi: 10.1002/clc.23319 (PMC7144483; doi:10.1002/clc.23319)
Supplement: Supplementary file 3 — Table S3 Prognostic role of sleep study parameters in univariate analysis [file CLC-43-329-s003.docx]

Supplemental Table 3 Prognostic role of sleep study parameters in univariate analysis

|  | Univariate Cox regression analysis | |
| --- | --- | --- |
|  | HR (95%CI) | *P* |
| AHI (per 1/h increase) | 1.003 (0.944-1.012) | 0.491 |
| AHI ≥ 15/h | 1.147 (0.859-1.532) | 0.354 |
| ODI (per 1/h increase) | 1.001 (0.993-1.010) | 0.751 |
| ODI ≥ 19.0/h | 1.105 (0.828-1.476) | 0.497 |
| MeanSO_2_ (per 1% increase) | 0.968 (0.924-1.013) | 0.159 |
| MeanSO_2_ < 95.0% | 1.124 (0.842-1.500) | 0.428 |
| MinSO_2_ (per 1% increase) | 0.995 (0.983-1.006) | 0.355 |
| MinSO_2_ < 79.0% | 1.289 (0.963-1.725) | 0.088 |
| T90% (per 1% increase) | 1.007 (1.000-1.014) | 0.049 |
| T90% ≥ 3.6% | 1.397 (1.045-1.869) | 0.024 |

AHI, the apnea-hypopnea index; CI confidence interval; HR, hazard ratio; MeanSO_2_, the mean oxygen saturation; MinSO_2_, the minimal oxygen saturation; ODI, oxygen desaturation index; T90%, the percentage of time with oxygen saturation below 90%
