# Supplementary material for: Prognostic value of sleep apnea and nocturnal hypoxemia in patients with decompensated heart failure
Source: Clin Cardiol. 2020 Jan 22;43(4):329–37. doi: 10.1002/clc.23319 (PMC7144483; doi:10.1002/clc.23319)
Supplement: Supplementary file 4 — Table S4 Factors selected in stepwise multivariate Cox regression analysis [file CLC-43-329-s004.docx]

Supplemental Table 4 Factors selected in stepwise multivariate Cox regression analysis

|  | Univariate Cox regression analysis | |
| --- | --- | --- |
|  | HR (95%CI) | *P* |
| Age (per 10 years increase) | 1.203 (1.091-1.326) | <0.001 |
| BMI (per 5 kg/m^2^ increase) | 0.509 (0.370-0.702) | <0.001 |
| Hypertension | 0.774 (0.579-1.034) | 0.083 |
| Renal dysfunction | 2.045 (1.524-2.744) | <0.001 |
| Atrial fibrillation | 1.316 (0.975-1.778) | 0.073 |
| NYNA Ⅲ/Ⅳ | 2.397 (1.520-3.778) | <0.001 |
| NTproBNP (per 500 pg/ml increase) | 1.038 (1.029-1.047) | <0.001 |
| LVEF (per 5% increase) | 0.941 (0.892-0.992) | 0.023 |
| MAP at discharge (per 10 mmHg increase) | 0.690 (0.583-0.815) | <0.001 |
| ACEI/ARB at discharge | 0.509 (0379-0.682) | <0.001 |
| Diuretics at discharge | 2.348 (1.103-5.000) | 0.027 |

ACEI, angiotensin converting enzyme inhibitor; ARB, angiotensin receptor blocker; BMI, body mass index; BUN, blood urea nitrogen; eGFR, estimated glomerular filtration rate; HbA1c, glycosylated hemoglobin; LVEF, left ventricular ejection fraction; MAP, mean arterial blood pressure; NT-proBNP, N-terminal pro-brain natriuretic peptide; NYHA, New York Heart Association;
