# Supplementary material for: Prognostic value of sleep apnea and nocturnal hypoxemia in patients with decompensated heart failure
Source: Clin Cardiol. 2020 Jan 22;43(4):329–37. doi: 10.1002/clc.23319 (PMC7144483; doi:10.1002/clc.23319)
Supplement: Supplementary file 5 — Table S5A, B, and C shows the process of multivariate Cox regression analysis, which are considered for review but not for publication [file CLC-43-329-s005.docx]

*Supplemental Table 5A, 5B and 5C shows the process of multivariate Cox regression analysis, which are considered for review but not for publication

Supplemental Table 5A

Stepwise multivariate proportional Cox regression analysis including T90%

|  | Univariate | | Multivariate | |
| --- | --- | --- | --- | --- |
|  | HR (95%CI) | *P* | HR (95%CI) | *P* |
| *T90% as a continuous variable* | | | | |
| Age (per 10 years increase) | 1.203 (1.091-1.326) | <0.001 | 1.144 (1.035-1.264) | 0.008 |
| BMI (per 5 kg/m^2^ increase) | 0.509 (0.370-0.702) | <0.001 | 0.630 (0.444-0.893) | 0.009 |
| Hypertension | 0.774 (0.579-1.034) | 0.083 | - | - |
| Renal dysfunction | 2.045 (1.524-2.744) | <0.001 | - | - |
| Atrial fibrillation | 1.316 (0.975-1.778) | 0.073 | - | - |
| NYNA Ⅲ/Ⅳ | 2.397 (1.520-3.778) | <0.001 | 1.672 (1.047-2.669) | 0.031 |
| NTproBNP (per 500 pg/ml increase) | 1.038 (1.029-1.047) | <0.001 | 1.027 (1.018-1.037) | <0.001 |
| LVEF (per 5% increase) | 0.941 (0.892-0.992) | 0.023 | - | - |
| MAP (per 10 mmHg increase) | 0.690 (0.583-0.815) | <0.001 | 0.797 (0.673-0.944) | 0.009 |
| ACEI/ARB | 0.509 (0379-0.682) | <0.001 | - | - |
| Diuretics | 2.348 (1.103-5.000) | 0.027 | - | - |
| T90% (per 1% increase) | 1.007 (1.000-1.014) | 0.049 | 1.008 (1.001-1.016) | 0.033 |
| *T90% as a categorical variable* | | | | |
| Age (per 10 years increase) | 1.203 (1.091-1.326) | <0.001 | 1.138 (1.030-1.258) | 0.011 |
| BMI (per 5 kg/m^2^ increase) | 0.509 (0.370-0.702) | <0.001 | 0.636 (0.450-0.900) | 0.011 |
| Hypertension | 0.774 (0.579-1.034) | 0.083 | - | - |
| Renal dysfunction | 2.045 (1.524-2.744) | <0.001 | - | - |
| Atrial fibrillation | 1.316 (0.975-1.778) | 0.073 | - | - |
| NYNA Ⅲ/Ⅳ | 2.397 (1.520-3.778) | <0.001 | 1.640 (1.026-2.622) | 0.039 |
| NTproBNP (per 500 pg/ml increase) | 1.038 (1.029-1.047) | <0.001 | 1.028 (1.019-1.038) | <0.001 |
| LVEF (per 5% increase) | 0.941 (0.892-0.992) | 0.023 | - | - |
| MAP (per 10 mmHg increase) | 0.690 (0.583-0.815) | <0.001 | 0.795 (0.671-0.943) | 0.008 |
| ACEI/ARB | 0.509 (0379-0.682) | <0.001 | - | - |
| Diuretics | 2.348 (1.103-5.000) | 0.027 | - | - |
| T90% ≥ 3.6% | 1.397 (1.045-1.869) | 0.024 | 1.408 (1.030-1.925) | 0.032 |

Supplemental Table 5B

Stepwise multivariate proportional Cox regression analysis including MinSO_2_

|  | Univariate | | Multivariate | |
| --- | --- | --- | --- | --- |
|  | HR (95%CI) | *P* | HR (95%CI) | *P* |
| *MinSO_2_ as a continuous variable* | | | | |
| Age (per 10 years increase) | 1.203 (1.091-1.326) | <0.001 | 1.147 (1.037-1.269) | 0.008 |
| BMI (per 5 kg/m^2^ increase) | 0.509 (0.370-0.702) | <0.001 | 0.652 (0.468-0.908) | 0.011 |
| Hypertension | 0.774 (0.579-1.034) | 0.083 | - | - |
| Renal dysfunction | 2.045 (1.524-2.744) | <0.001 | - | - |
| Atrial fibrillation | 1.316 (0.975-1.778) | 0.073 | - | - |
| NYNA Ⅲ/Ⅳ | 2.397 (1.520-3.778) | <0.001 | 1.774 (1.112-2.831) | 0.016 |
| NTproBNP (per 500 pg/ml increase) | 1.038 (1.029-1.047) | <0.001 | 1.028 (1.019-1.038) | <0.001 |
| LVEF (per 5% increase) | 0.941 (0.892-0.992) | 0.023 | - | - |
| MAP (per 10 mmHg increase) | 0.690 (0.583-0.815) | <0.001 | 0.780 (0.660-0.922) | 0.004 |
| ACEI/ARB | 0.509 (0379-0.682) | <0.001 | - | - |
| Diuretics | 2.348 (1.103-5.000) | 0.027 | - | - |
| MinSO_2_ (per 1% increase) | 0.995 (0.983-1.006) | 0.355 | 0.985 (0.973-0.997) | 0.017 |
| *MinSO_2_ as a categorical variable* | | | | |
| Age (per 10 years increase) | 1.203 (1.091-1.326) | <0.001 | 1.147 (1.037-1.268) | 0.007 |
| BMI (per 5 kg/m^2^ increase) | 0.509 (0.370-0.702) | <0.001 | - | - |
| Hypertension | 0.774 (0.579-1.034) | 0.083 | - | - |
| Renal dysfunction | 2.045 (1.524-2.744) | <0.001 | - | - |
| Atrial fibrillation | 1.316 (0.975-1.778) | 0.073 | - | - |
| NYNA Ⅲ/Ⅳ | 2.397 (1.520-3.778) | <0.001 | 1.782 (1.117-2.845) | 0.015 |
| NTproBNP (per 500 pg/ml increase) | 1.038 (1.029-1.047) | <0.001 | 1.028 (1.018-1.037) | <0.001 |
| LVEF (per 5% increase) | 0.941 (0.892-0.992) | 0.023 | - | - |
| MAP (per 10 mmHg increase) | 0.690 (0.583-0.815) | <0.001 | 0.789 (0.667-0.934) | 0.006 |
| ACEI/ARB | 0.509 (0379-0.682) | <0.001 | 0.665 (0.489-0.906) | 0.010 |
| Diuretics | 2.348 (1.103-5.000) | 0.027 | - | - |
| MinSO_2_ < 79.0% | 1.289 (0.963-1.725) | 0.088 | 1.395 (1.038-1.876) | 0.028 |

Supplemental Table 5C

Stepwise multivariate proportional Cox regression analysis including MeanSO_2_

|  | Univariate | | Multivariate | |
| --- | --- | --- | --- | --- |
|  | HR (95%CI) | *P* | HR (95%CI) | *P* |
| *MeanSO_2_ as a continuous variable* | | | | |
| Age (per 10 years increase) | 1.203 (1.091-1.326) | <0.001 | 1.127 (1.019-1.247) | 0.020 |
| BMI (per 5 kg/m^2^ increase) | 0.509 (0.370-0.702) | <0.001 | 0.636 (0.449-0.900) | 0.011 |
| Hypertension | 0.774 (0.579-1.034) | 0.083 | - | - |
| Renal dysfunction | 2.045 (1.524-2.744) | <0.001 | - | - |
| Atrial fibrillation | 1.316 (0.975-1.778) | 0.073 | - | - |
| NYNA Ⅲ/Ⅳ | 2.397 (1.520-3.778) | <0.001 | 1.671 (1.046-2.669) | 0.032 |
| NTproBNP (per 500 pg/ml increase) | 1.038 (1.029-1.047) | <0.001 | 1.029 (1.020-1.039) | <0.001 |
| LVEF (per 5% increase) | 0.941 (0.892-0.992) | 0.023 | - | - |
| MAP (per 10 mmHg increase) | 0.690 (0.583-0.815) | <0.001 | 0.800 (0.675-0.947) | 0.010 |
| ACEI/ARB | 0.509 (0379-0.682) | <0.001 | - | - |
| Diuretics | 2.348 (1.103-5.000) | 0.027 | - | - |
| MeanSO_2_ (per 1% increase) | 0.968 (0.924-1.013) | 0.159 | 0.950 (0.905-0.998) | 0.040 |
| *MeanSO_2_ as a categorical variable* | | | | |
| Age (per 10 years increase) | 1.203 (1.091-1.326) | <0.001 | 1.144 (1.036-1.265) | 0.008 |
| BMI (per 5 kg/m^2^ increase) | 0.509 (0.370-0.702) | <0.001 | - | - |
| Hypertension | 0.774 (0.579-1.034) | 0.083 | - | - |
| Renal dysfunction | 2.045 (1.524-2.744) | <0.001 | - | - |
| Atrial fibrillation | 1.316 (0.975-1.778) | 0.073 | - | - |
| NYNA Ⅲ/Ⅳ | 2.397 (1.520-3.778) | <0.001 | 1.714 (1.075-2.734) | 0.024 |
| NTproBNP (per 500 pg/ml increase) | 1.038 (1.029-1.047) | <0.001 | 1.029 (1.020-1.039) | <0.001 |
| LVEF (per 5% increase) | 0.941 (0.892-0.992) | 0.023 | - | - |
| MAP (per 10 mmHg increase) | 0.690 (0.583-0.815) | <0.001 | 0.793 (0.670-0.938) | 0.007 |
| ACEI/ARB | 0.509 (0379-0.682) | <0.001 | 0.698 (0.514-0.948) | 0.021 |
| Diuretics | 2.348 (1.103-5.000) | 0.027 | - | - |
| MeanSO_2_ < 95.0% | 1.124 (0.842-1.500) | 0.428 | - | - |
